# Education and training of clinical research professionals and the evolution of the Joint Task Force for Clinical Trial Competency

**DOI:** 10.3389/fphar.2024.1291675

**Published:** 2024-01-18

**Authors:** Stephen A. Sonstein, Honorio Silva, Carolynn T. Jones, Barbara E. Bierer

**Affiliations:** ^1^ Multi-Regional Clinical Trials Center of Brigham and Women’s Hospital and Harvard, Boston, MA, United States; ^2^ Academy of Global Medicines Development Professionals, New York, NY, United States; ^3^ King’s College London, London, United Kingdom; ^4^ College of Nursing, and Center for Clinical Translational Sciences, The Ohio State University, Columbus, OH, United States; ^5^ Division of Global Health Equity, Department of Medicine, Brigham and Women’s Hospital and Harvard Medical School, Boston, MA, United States

**Keywords:** Joint Task Force for Clinical Trial Competency, accreditation, academic programs in clinical research, clinical research professional, clinical research workforce, pharmaceutical physician

## Abstract

Clinical research professionals play a critical role in the design, conduct, and oversight of clinical trials, and they must have the knowledge, skills, and abilities to ensure that trials are conducted ethically, safely, and in accordance with regulatory requirements. As clinical research has evolved from being a necessary activity for the development and regulatory approval of new medicines to an accredited academic discipline and, more recently, to a globally recognized profession, the methods of education and training of professionals have also evolved. Initially, on-the-job informal coaching and specialized training organizations led to formalized and accredited academic degree programs and, more recently, to international competency standards and competency maintenance through continuous professional development. The Joint Task Force (JTF) for Clinical Trial Competency is a multidisciplinary, international group of experts who came together to aggregate and refine competency standards for clinical research professionals, first published in 2014. The 8 domains and 49 specific core competencies of the JTF Framework have become a globally recognized standard upon which education and training programs, role descriptions, and upward mobility criteria for professionals are now based. The JTF meets regularly and, through its workgroups, continues to evolve in response to the changing needs of the profession. The JTF is committed to continuous improvement to ensure that clinical research professionals have the competence necessary to conduct safe, ethical, and high-quality clinical research.

## 1 Introduction

Clinical research is the bedrock of advancements in diagnosis, treatments, and procedures to improve the public health. Beginning with early discovery work, leading to human trials and, ultimately, to regulatory marketing approvals of products to the public, the teams that assemble to accomplish this work constitute a complex network of experts and professionals. Professional pathways for basic, translational, and clinical sciences have been defined for principal investigators, doctoral trainees, and pharmaceutical physicians (Nathan, 2002; Meyers et al., 2012; Silva et al., 2013). However, the professional pathways for the large number of staff that support the various activities required for operationalizing and managing clinical research studies are generally less defined and vary with the local definition of the role. At a clinical research site, such clinical research professional (CRP) staff roles include clinical research assistants, clinical research coordinators, or specialized areas such as data managers, quality compliance officers, and regulatory affairs specialists. At the sponsor level, pharmaceutical physicians may lead research and regulatory strategies for the development of new potential targets. Individuals working as CRPs for pharmaceutical sponsors or contract research organizations may perform roles such as site monitors, data managers, safety officers, and project leads. CRP staff may have a wide variety of educational backgrounds with associates degrees, diplomas and graduate degrees, and specific competency-based training or additional academic education in clinical research. A pharmaceutical physician is a medicine development role that requires a baccalaureate degree, an MD and licensure, or a PharmD, who has an additional diploma education in pharmaceutical medicine (Silva et al., 2013). Advancement pathways for these individuals working at the research site and in the pharmaceutical industry are beginning to be better defined; however, in today’s workforce climate, severe staff shortages threaten to slow clinical research progress (Freel et al., 2023). The professionalization of the clinical research workforce is dependent on the early recognition of the professional roles and their importance and competency standards defining the work, educational pathways, and professional development paths so that the pool of individuals interested in this work are aware of the opportunities in this field.

### 1.1 Defining competency standards

The United States National Institutes of Health (NIH), National Center for Advancing Translational Sciences (NCATS), has focused their efforts on expanding the clinical research workforce (Committee et al., 2013). The United States National Academies of Science, Engineering, and Medicine (NASEM, formerly the American Institute of Medicine) called for innovation in the clinical trial enterprise, suggesting the closer integration of healthcare delivery with clinical trials (Califf et al., 2012). Early publications on clinical research skills began to emerge, and publications on competencies for clinical research nurses were published by the United Kingdom Royal College of Nursing (UK Clinical Research Collaboration Subcommittee for Nurses in Clinical Research, 2011), the United States National Institutes of Health Clinical Center (CRN, 2010 Domain of Practice Committee, 2009), and the Oncology Nursing Society (Oncology Nursing Society, 2010). The Association of Clinical Research Professionals (ACRP) began to build a set of targeted trainings; NCATS published competencies for investigators (NCATS. Core, 2011), and knowledge, skills, and abilities (KSAs) for pharmaceutical physicians were also published by the International Federation of Associations of Pharmaceutical Physicians (IFAPP) (Silva et al., 2013). A collaboration among these groups and others initially came together to synthesize the available literature and consolidate skillsets into a set of KSAs that would strengthen the educational curricula for CRPs (Jones et al., 2012). Subsequently, several organizations, many of which included individuals who directed and taught in academic programs in clinical research and had extensive experience working across multiple sectors of the clinical research enterprise, began to outline the standards necessary to perform clinical research. This working group consisted of members from the ACRP, Association of Pharmaceutical Physicians and Investigators (APPI), Consortium of Academic Programs in Clinical Research (CoAPCR), United States NIH Clinical Translational Science Award (CTSA) program, Global Health Network (GHN), IFAPP, Multi-Regional Clinical Trials Center (MRCT Center) of Brigham and Women’s Hospital and Harvard, and TransCelerate BioPharma.

### 1.2 Launching the JTF framework

**FIGURE 1:**
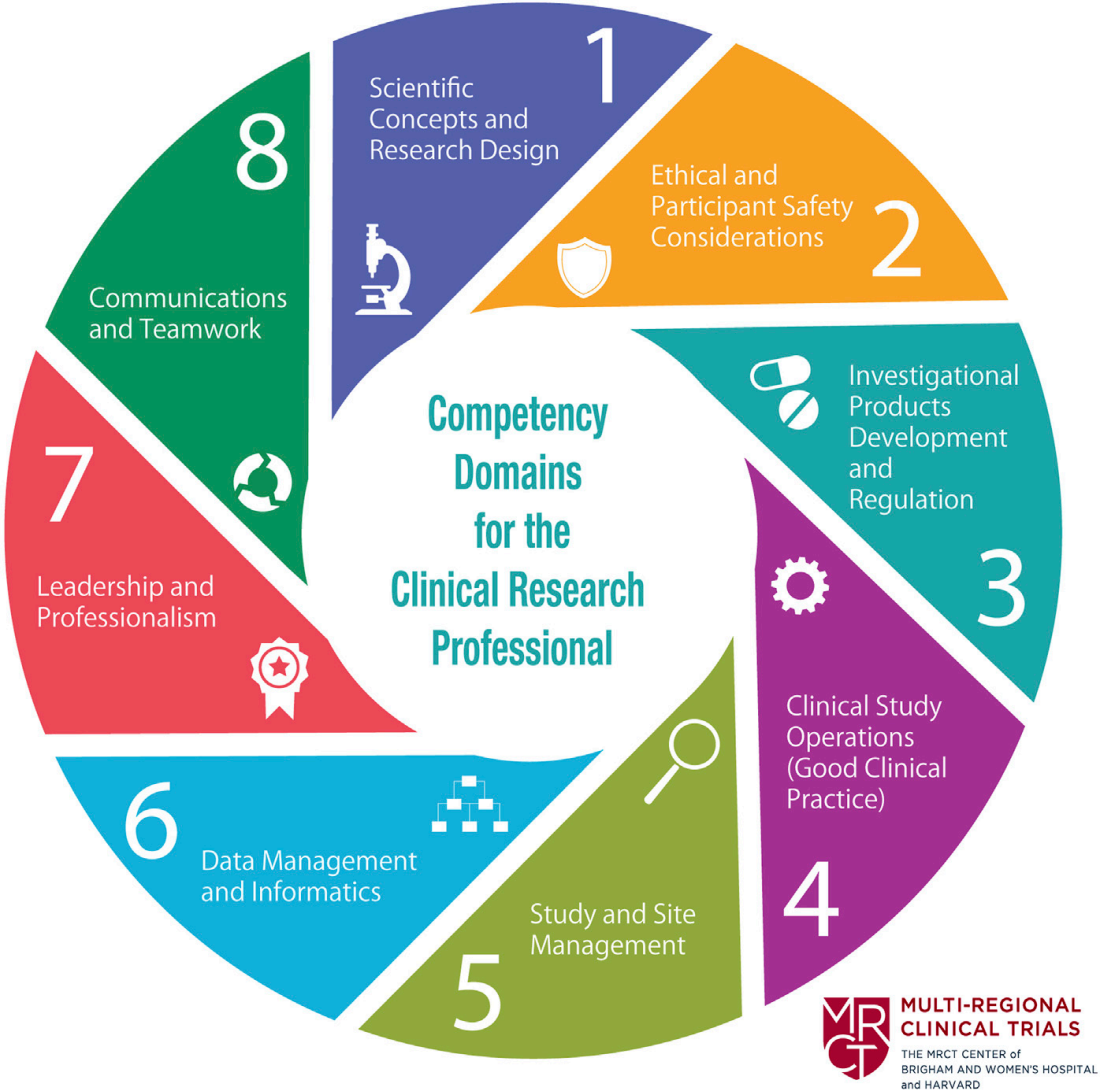
Competency domains for the clinical research professional.

In 2013, a meeting was organized by the MRCT Center in collaboration with 18 other organizations and institutions to address issues related to the training of clinical research professionals. At the meeting, several individuals noted that there were no defined and globally recognized competency standards for clinical research professionals, despite the definition by the International Council on Harmonisation (ICH) of Technical Requirements for Pharmaceuticals for Human Use of good clinical practices (GCPs) and the expectation of appropriate training and competencies by global regulatory agencies. This diverse group of representatives from the pharmaceutical industry, academic educational programs, clinical sites, and contract research organizations agreed to review the literature concerning competency standards for the various clinical research roles and to align them to a global set of competency standards to reflect the needs of the clinical research enterprise. The group named itself the Joint Task Force (JTF) for Clinical Trial Competency, and members aligned and harmonized the many role-based competencies from the published literature into the JTF Clinical Trial Core Competency Framework (JTF Framework) Version 1.0, which represented the competencies of the entire clinical research workforce. The framework consisted of eight domains: 1) Scientific Concepts and Research Design; 2) Ethical and Participant Safety Considerations; 3) Investigational Product Development and Regulation; 4) Clinical Study Operations; 5) Study and Site Management; 6) Data Management and Informatics; 7) Leadership and Professionalism; and 8) Communication and Teamwork. Each domain included multiple harmonized and related competencies (51 competencies in all). The framework was first published in 2014 (Figure 1) (Sonstein et al., 2014).

The publication of the JTF Framework was supplemented by presentations and other forms of dissemination, describing the multiple ways in which the JTF Framework could be applied. In 2015, the JTF conducted a global survey of the clinical research workforce, including investigators, clinical coordinators, regulatory affairs professionals, research managers, data managers, and clinical trial monitors to validate the applicability of the JTF Core Competency Framework to assess the self-perceived competency level across the JTF domains by role and to inform the enterprise of the education and training needs for each role. Significant gaps were revealed in domain 1 (Scientific Concepts and Research Design) and domain 3 (Investigational Products Development and Regulation), a finding that contributed to future academic and training initiatives (Sonstein et al., 2016). The JTF Competency Framework was adopted by clinical research professional associations, ACRP, and the Society of Clinical Research Associates (SoCRA) and incorporated into revised certification examination content and training programs. These competencies were also adopted by academic researchers who later expanded the JTF Framework to include competencies for investigators and coordinators conducting not only clinical trials but also social, behavioral, and community research (Calvin-Naylor et al., 2017; Murphy et al., 2018). NCATS funded a multi-institutional effort to collect trainings relevant to each of the competencies, culminating in a freely available portal that enabled access to those educational opportunities (Ianni et al., 2020). Version 2.0 of the JTF competencies was the result of an editorial review that combined redundant competencies resulting in a total of 47 competencies for the 8 original domains. At the clinical site level, Duke University revised their job titles and job descriptions around the JTF competencies, and onboarding programs were redesigned to align with the JTF competencies (Brouwer et al., 2016; Saunders et al., 2017). Academic programs in clinical research through the CoAPCR endorsed the JTF competencies, incorporated them into educational curricula, and applied them to develop accreditation standards for educational programs in clinical research. Subsequently, the Commission on Accreditation of Allied Health Education Programs (CAAHEP) agreed to utilize the JTF Framework as the basis for formal academic program accreditation and supported the formation of the Committee on Accreditation of Academic Programs in Clinical Research (CAAPCR) (Commission on Accreditation of Allied Health Programs, 2012; Sonstein and Jones, 2018). In tandem with these educational initiatives, IFAPP further developed KSAs of core competencies and proposed the alignment of those to educational content for pharmaceutical physicians, among others involved in medicine development (Silva et al., 2015; Stonier et al., 2020).

### 1.3 Leveling the JTF Framework: calibrating experience, understanding, and expertise

The JTF recognized that professional competency evolved with experience and education. A working group was formed that consisted of advanced clinical research professionals who were leaders across site- and sponsor-related sectors and included international representation. The group agreed to align the specific competencies by experience and expertise as 1) fundamental, defined as can perform the task/and exhibit the knowledge at an essential or fundamental level; may require some coaching or supervision; 2) skilled, defined as can perform task or skill independently, navigate resources, and use tools well; and 3) advanced, defined as demonstrates advanced skills and knowledge and the ability to teach, coach, or supervise others; consistently applies critical thinking and problem solving (Sonstein et al., 2020). The working group was divided into five smaller sub-groups that were charged with creating a first-round set of leveled and measurable competency statements, with examples. Using a modified Delphi approach, these competency statements and examples were rotated amongst the other sub-groups, whereby they determined whether to keep the leveled competencies and examples and if so, edit them. When the leveled competencies and examples for each of the eight domains completed the cycle, the groups did a second-level cycle, making track-change edits. Ultimately, the chairs of each sub-group conducted the final synthesis and edits for publication. The resulting leveled competencies were presented as a new version 3.0 of the JTF Clinical Trial Competency Framework, one that maintained the 8 domains but expanded the numbers of measurable competencies, with each competency having an average of 3–5 additional measurable, leveled skillsets (Sonstein et al., 2020). Some institutions relied on this work to develop institutional job descriptions that were consistent in their expectations with respect to experience and salary grade, contributing to the development of a tiered career progression pathway for clinical research professionals that helped improve CRP turnover rates (Stroo et al., 2020).

### 1.4 Expanding clinical research roles reflected in the JTF Framework

In 2019, members of the project management communities in clinical research noted that the original JTF Framework did not specifically include project management competencies. A new JTF working group was charged with the task of suggesting appropriate additions to reflect the competencies of the project and program managers. This led to the addition of two additional core competencies, bringing the number of core competencies to 49 (Sonstein et al., 2022a). This current version 3.1 of the framework can be found on the MRCT JTF website (Joint Task Force for Clinical Trial Competency, 2022).

### 1.5 International reach of the JTF Framework

The awareness and relevance of the JTF Framework has continued to expand internationally. Increasingly, educational programs, onboarding programs, and professional development efforts have been based upon and incorporated into the JTF Framework. Moreover, as the JTF Framework has become a globally recognized resource, new translations of the framework into Spanish, Japanese, French, Thai, Bahasa Indonesia, Italian, Vietnamese, Chinese, and Korean have been made publicly available (Joint Task Force for Clinical Trial Competency, 2022). Additional translations are in progress.

## 2 Discussion

This manuscript summarizes the decade-long evolution and impact of the JTF Framework on the education and professionalization of CRPs, investigators and their study teams, pharmaceutical, and other professionals involved in medicine development and clinical research. Professionalizing the workforce is a strategic goal for the clinical research enterprise as shortages in the workforce threaten operations and clinical trial progress (Freel et al., 2023). The basic content for clinical research professional academic curricula, training curricula, job titles, professional advancement, ePortfolios, professional certification, research, team science competencies for CRPs, competency assessments, and international applications are all influenced by the adoption and application of the JTF Framework (Association of Clinical Research Professionals, 2018; Stonier et al., 2020; Ivey, 2021; Jones et al., 2021; Society of Clinical Research Associates; Sonstein et al., 2022b; Glaettli et al., 2022; Ibrahim et al., 2022; Mendell et al., 2023). A CTSA working group has conducted a leveled approach to define CRP team science competencies, which provides more granularity to JTF domains 7 and 8 covering Leadership, Professionalism, Communication, and Teamwork (Mendell et al., 2023). Additionally, because of the emerging technology and requirements in data management (Ittenbach, 2023), future work is underway to address expanding data management competencies, leveled by necessary skills, for today’s digital era. In conclusion, having attained global recognition, the JTF Framework is an important resource to educators, trainers, and clinical research leadership and management. The JTF Framework will continue to evolve in response to the rapidly changing clinical research enterprise and will continue to be integrated into international clinical research structures. The JTF Framework will contribute to strengthen the workforce, enhance clinical research operations, and empower a professional identity that is essential for public health.

## 3 Scope statement

This perspective article meets the special topic collection entitled Building the Clinical Research Workforce: Challenges, Capacities, and Competencies. Here, we summarize the decade-long contribution of the JTF Clinical Trial Core Competency Framework that defined the educational standards and competencies for the clinical research workforce, especially clinical research professionals working at clinical research sites, contract research organizations, and sponsors. The application of this framework has had broad and international impact. As a living competency framework, the JTF competencies continue to adapt to emerging trends in clinical research.

## Data Availability

The original contributions presented in the study are included in the article/Supplementary Material; further inquiries can be directed to the corresponding author.
